# Early antiretroviral therapy and daily pre‐exposure prophylaxis for HIV prevention among female sex workers in Cotonou, Benin: a prospective observational demonstration study

**DOI:** 10.1002/jia2.25208

**Published:** 2018-11-22

**Authors:** Aminata Mboup, Luc Béhanzin, Fernand A Guédou, Nassirou Geraldo, Ella Goma‐Matsétsé, Katia Giguère, Marlène Aza‐Gnandji, Léon Kessou, Mamadou Diallo, René K Kêkê, Moussa Bachabi, Kania Dramane, Lily Geidelberg, Fiona Cianci, Christian Lafrance, Dissou Affolabi, Souleymane Diabaté, Marie‐Pierre Gagnon, Djimon M Zannou, Flore Gangbo, Marie‐Claude Boily, Peter Vickerman, Michel Alary

**Affiliations:** ^1^ Département de médecine sociale et préventive Université Laval Québec QC Canada; ^2^ Axe Santé des populations et pratiques optimales en santé Centre de recherche du CHU de Québec – Université Laval Québec QC Canada; ^3^ Dispensaire IST Centre de santé communal de Cotonou 1 Cotonou Bénin; ^4^ École Nationale de Formation des Techniciens Supérieurs en Santé Publique et en Surveillance Épidémiologique Université de Parakou Parakou Bénin; ^5^ Service de Consultance et Expertise Nouvelle en Afrique (SCEN AFRIK) Cotonou Bénin; ^6^ Programme Santé de Lutte contre le Sida (PSLS) Cotonou Bénin; ^7^ Laboratoire de virologie du Centre Muraz Bobo‐Dioulasso Burkina Faso; ^8^ Department of infectious disease Imperial College London London UK; ^9^ University of Bristol Bristol UK; ^10^ London School of Hygiene and Tropical Medicine London UK; ^11^ Faculté des sciences de la santé Université d'Abomey‐Calavi Cotonou Bénin; ^12^ Centre national hospitalier universitaire HMK de Cotonou Cotonou Bénin; ^13^ Université Alassane Ouattara Bouaké Côte d'Ivoire; ^14^ Faculté des sciences infirmières Université Laval Québec QC Canada; ^15^ Institut national de santé publique du Québec Québec QC Canada

**Keywords:** HIV prevention, early antiretroviral therapy, female sex workers, pre‐exposure prophylaxis, retention, adherence

## Abstract

**Introduction:**

In sub‐Saharan Africa, HIV prevalence remains high, especially among key populations. In such situations, combination prevention including clinical, behavioural, structural and biological components, as well as adequate treatment are important. We conducted a demonstration project at the Dispensaire IST, a clinic dedicated to female sex workers (FSWs) in Cotonou, on early antiretroviral therapy (E‐ART, or immediate “test‐and‐treat”) and pre‐exposure prophylaxis (PrEP). We present key indicators such as uptake, retention and adherence.

**Methods:**

In this prospective observational study, we recruited FSWs from October 4th 2014 to December 31st 2015 and followed them until December 31st 2016. FSWs were provided with daily tenofovir disoproxil fumarate/emtricitabine (Truvada^®^) for PrEP or received a first‐line antiretroviral regimen as per Benin guidelines. We used generalized estimating equations to assess trends in adherence and sexual behaviour.

**Results:**

Among FSWs in the catchment area, HIV testing coverage within the study framework was 95.5% (422/442). At baseline, HIV prevalence was 26.3% (111/422). Among eligible FSWs, 95.5% (105/110) were recruited for E‐ART and 88.3% (256/290) for PrEP. Overall retention at the end of the study was 59.0% (62/105) for E‐ART and 47.3% (121/256) for PrEP. Mean (±SD) duration of follow‐up was 13.4 (±7.9) months for E‐ART and 11.8 (±7.9) months for PrEP. Self‐reported adherence was over 90% among most E‐ART participants. For PrEP, adherence was lower and the proportion with 100% adherence decreased over time from 78.4% to 56.7% (*p*‐trend < 0.0001). During the 250.1 person‐years of follow‐up among PrEP initiators, two seroconversions occurred (incidence 0.8/100 person‐years (95% confidence interval: 0.3 to 1.9/100 person‐years)). The two seroconverters had stopped using PrEP for at least six months before being found HIV‐infected. In both groups, there was no evidence of reduced condom use.

**Conclusions:**

This study provides data on key indicators for the integration of E‐ART and PrEP into the HIV prevention combination package already offered to FSWs in Benin. PrEP may be more useful as an individual intervention for adherent FSWs rather than a specific public health intervention. E‐ART was a more successful intervention in terms of retention and adherence and is now offered to all key populations in Benin.

**Study registration:**

ClinicalTrials.gov NCT02237

## 1 Introduction

In sub‐Saharan Africa, despite all the preventive and therapeutic efforts accomplished so far, the HIV epidemic still persists in key populations 1. In 2016, approximately 64% of the 5000 new HIV infections occurring worldwide daily occurred in that region 2. In West Africa, studies have shown that female sex workers (FSWs) have historically contributed directly or indirectly to 52% to 90% of HIV transmission cases towards the general population essentially via their clients and other male sexual partners 3, 4, 5. Thus, preventing HIV among FSWs has the potential to have population level public health impact. The burden of HIV remains disproportionately high among FSWs in this region despite substantial prevention efforts in the last years and reductions in the prevalence of HIV and other sexually transmitted infections (STIs) 6, 7, 8. FSWs are reported to have an HIV prevalence over 12 times higher than other women of reproductive age in sub‐Saharan Africa 9. In Benin, in 2015, HIV prevalence was estimated at 1.0 (95% confidence interval (CI): (0.7 to 1.4)) in the general population and at 15.7% (95% CI: (13.7 to 17.7)) among FSWs 10.

In such situations, it is important to tailor combination prevention, including clinical, behavioural, structural and biological components, for the sex work milieu in general and for FSWs in particular, in addition to ensuring adequate treatment 11. Recent research has shown that pre‐exposure prophylaxis (PrEP, the use of antiretroviral drugs by HIV seronegative individuals) and early antiretroviral therapy (E‐ART) for all infected individuals regardless of CD4 count are promising interventions to prevent HIV acquisition and transmission in high risk populations if uptake and treatment adherence are high 12, 13, 14. However, randomized trials have shown disappointing results for PrEP among women at high risk in African countries because of low adherence 15, 16. It is thus essential to evaluate the effectiveness of these interventions in “real‐world” settings since it may vary based on personal, social and cultural characteristics. Although studies assessing the feasibility of these two new prevention methods in “real‐world” settings are ongoing 17 or have been recently completed 18, limited data are available on their implementation among FSWs in the West African region, especially in countries where sex work is not legalized like Benin. We thus conducted this demonstration project to assess the feasibility and usefulness of adding E‐ART (immediate “test‐and‐treat”) and PrEP to the combination prevention package, including clinical, behavioural and structural components, already offered to FSWs in Benin. In this paper, we are presenting primary results on uptake, retention and adherence.

## 2 Methods

### 2.1 Study settings

This prospective observational cohort study was conducted in Cotonou, the largest city in Benin, and its inner suburbs among “professional” FSWs (women whose revenue is mostly generated by sex work). The study was carried out at the Dispensaire IST (DIST), a FSW‐friendly clinic providing confidential clinical services and free HIV/STI treatment to FSWs in Cotonou and supported by the National AIDS Control Program. The catchment area and sample size were defined by mapping and enumeration of “professional” FSWs in Cotonou. We expected to reach about 500 FSWs and recruit and follow‐up at least 90% of the HIV‐positive FSWs (n~100) on E‐ART and at least 60% of the HIV‐negative FSWs (n~250) on PrEP. More details are presented in Text S1.

### 2.2 Study design

FSWs from the pre‐defined catchment area in Cotonou were informed about the study and invited for a screening visit at the DIST by community workers. Recruitment in the study was planned to spread from downtown Cotonou towards the suburbs. When a saturation point was reached in one area, we recruited in another neighbouring area towards outskirts till the planned sample size was achieved. At the screening visit, participants’ HIV status and eligibility criteria were assessed. HIV rapid tests were performed. Additional tests such as hepatitis B and syphilis serologies, renal and liver function tests, and for HIV‐positive women, CD4 count and viral load were carried out. HIV status was confirmed at recruitment (10 to 14 days after screening) using additional HIV tests performed on separate blood samples. Women who agreed to participate underwent gynaecological exams during which swabs were collected for direct microscopy and for testing for genital gonococcal and chlamydial infections with nucleic acid amplification tests. Laboratory procedures are presented in Text S1. At the recruitment visit, participants were informed of the results from the screening visit and the implication of the results. This visit allowed potential enrolment into E‐ART for HIV‐positive FSWs or PrEP for HIV‐negative FSWs. Women on PrEP were provided with tenofovir disoproxil fumarate/emtricitabine (TDF/FTC, Truvada^®^) to be taken once a day and participants on E‐ART received a first‐line ART regimen as per Benin guidelines. The first follow‐up visit was scheduled 14 days after recruitment, to assess short‐term side effects and early adherence. The next follow‐up visit was scheduled at month 3, and subsequent visits were scheduled quarterly. Recruitment was carried out from 4 October 2014 to 31 December 2015, with an additional 12‐month follow‐up period till 31 December 2016; total follow‐up thus varied from 12 to 24 months depending on the time of recruitment. The medications were refilled every month, but participants could pick‐up a supply for up to three months if needed. Participants could resume follow‐up in the study even after missing a scheduled visit. Four community workers were trained to regularly remind study participants of their visits by telephone or by visiting them at home or their work place. Active tracking was carried out for those that missed an appointment. All participants were counselled on the importance of adherence and received free condoms at each follow‐up visit. Assistance for transportation was provided by community workers when needed, a practice that was already in place for standard quarterly clinical care for FSWs at the DIST prior to the study.

### 2.3 Eligibility criteria

Participants were eligible for E‐ART if they were 18 years or older, HIV‐positive at screening and reconfirmed on a second sample, and HIV treatment naive. Women with HIV‐2 and dual infection were not included in the E‐ART arm and were referred for treatment at the DIST. Pregnant and breastfeeding women were not excluded from the E‐ART arm and were referred to the prevention of mother‐to‐child transmission programme in Benin. For PrEP, participants were eligible if they were ≥18 years old and HIV‐negative at both screening and recruitment visits. Other exclusion criteria for PrEP were a compromised renal function (creatinine clearance <60 mL/min), active hepatitis B and/or abnormal liver function, pregnancy and breastfeeding.

### 2.4 Outcomes

We evaluated the feasibility of E‐ART and PrEP through a set of indicators, including screening coverage, uptake, adherence, retention in the cohort, condom migration (decrease in condom use as a result of being substituted by another preventive intervention) and development of drug resistance. Face‐to‐face interviews (FTFI) at the screening visit were used to collect data on socio‐demographic characteristics, HIV testing history and general health status. We collected information on the number of clients and other sexual partners, sexual practices, condom use, self‐reported adherence to ART, STI diagnoses and other general symptoms since last visit by FTFI. Case report forms were used to collect clinical and biological data. We evaluated coverage based on the number of all screened FSWs in the catchment area and uptake based on the number of eligible FSWs enrolled.

For E‐ART, self‐reported adherence was measured by asking participants the number of pills missed in the last month. For PrEP, participants were asked the number of pills missed in the last week. Blood samples were collected to evaluate TDF/FTC concentrations; results will be presented subsequently. For E‐ART participants, CD4 and viral load levels were assessed, the latter being a good marker of treatment adherence.

We monitored seroconversion through HIV rapid testing at each follow‐up visit among PrEP participants. Incidence rates for HIV and STIs were calculated by dividing the number of incident cases by the person time of follow‐up. Retention was evaluated at each study visit. Women who missed visits and came back later were included in the analysis. We assessed trends in sexual behaviour (number of clients having had sex with a regular partner and consistent, that is, 100% condom use with these two types of partners) in the last two and fourteen days.

### 2.5 Ethics

The study protocol was approved by the ethics committee of the CHU de Québec–Université Laval, Québec, Canada, and the Benin National Ethics Committee for Health Research. All participants provided written consent in a free and informed manner and could decline or withdraw from the study at any time. Participants received a financial compensation of 4000 FCFA (about $8) at each visit to compensate for their travel cost and the time they devoted to the study.

### 2.6 Statistical analysis

The Chi‐square and Student's *t*‐tests were used to compare proportions and means respectively, between E‐ART and PrEP participants. The analyses of time trends during the course of the study used generalized estimating equations with an unstructured matrix to take into account dependency between the observations within individuals. Statistical analyses were performed using SAS 9.4 (SAS Institute Inc, Cary, NC, USA). This study is registered with ClinicalTrials.gov, number NCT02237027.

## 3 Results

### 3.1 Coverage and uptake

**Figure 1:**
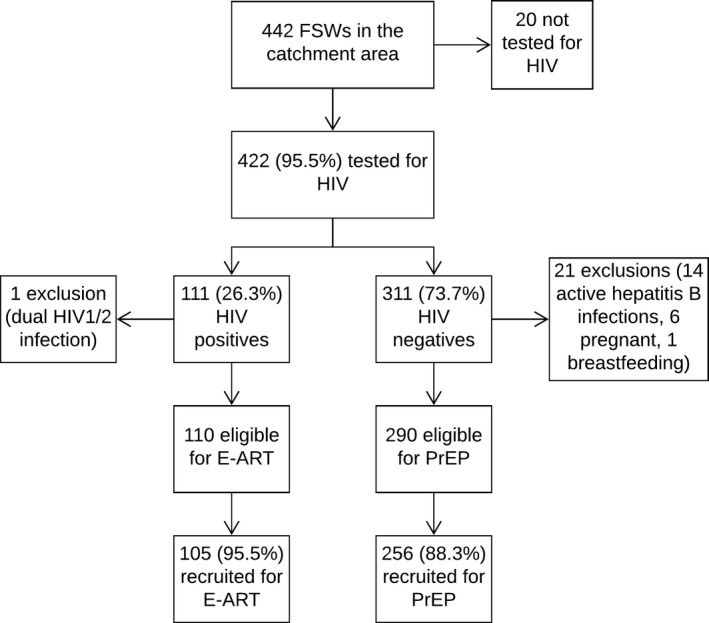
**Flow chart of screening coverage, HIV prevalence and E‐ART/PrEP uptake among FSWs in Cotonou, Benin (2014 to 2016)** The 442 FSWs worked in the catchment area and were not known to be ART‐treated HIV infected women. They could however be known as HIV‐infected but were not yet on ART at the time of recruitment in the study. ART, antiretroviral therapy; FSW, female sex workers; PrEP, pre‐exposure prophylaxis; E‐ART, early antiretroviral therapy.

Of the 442 FSWs working in the catchment area and not known to be ART‐treated HIV‐positive women, 95.5% (422/442) were tested for HIV. HIV prevalence was 26.3% (111/422). Among screened FSWs, 0.9% (1/111, due to dual HIV‐1/2 infection) and 6.8% (21/311, for various reasons) were ineligible respectively for E‐ART and PrEP. Among eligible women for E‐ART and PrEP, 95.5% (105/110) and 88.3% (256/290) were respectively recruited (*p *= 0.031). The study was closed on 31 December 2016. Figure 1 presents a flow chart of the different steps related to recruitment in the study. Table S1 shows reasons for non‐participation among eligible subjects.

### 3.2 Baseline characteristics

Table 1 shows the characteristics of participants at baseline. Median age of all participants was 32 years (interquartile range: 26 to 40). E‐ART participants were older than PrEP participants (*p *= 0.005). Half of the FSWs were Beninese, and half were from surrounding countries. About one‐third of all participants never attended school. E‐ART participants were less educated than PrEP participants (41.4% with no education vs. 29.3%; *p *= 0.037). Most FSWs were divorced/separated or widowed (64.5% or 233/361). At baseline, there was no case of active syphilis, but the prevalence of gonorrhea and chlamydia were 9.7% and 4.8% respectively. The prevalence of bacterial vaginosis, candidiasis and trichomoniasis were 59.8%, 4.8% and 0.6% respectively. Among all FSWs tested for HIV at screening, the prevalence of active hepatitis B was 4.5% and similar among both HIV‐positive and ‐negative women, whereas 69.9% were anti‐HBc positive (83.8% among HIV‐positive and 65.0% among HIV‐negative, *p *< 0.001), indicating the occurrence of past or current hepatitis B infection. Comparison of baseline characteristics of acceptors/decliners within the categories of E‐ART and PrEP are presented in Tables S2 and S3. Briefly, in both study groups, FSWs eligible but not recruited were younger and more often single than FSWs recruited.

**Table 1:** Baseline characteristics of FSWs recruited in an E‐ART‐PrEP demonstration project in Cotonou, Benin (2014 to 2016)

Characteristics	E‐ART N* *= 105 n (%)	PrEP N* *= 256 n (%)	Total N* *= 361 n (%)
Mean age^a^ (±SD) (years)	35.5 ± 8.8	32.5 ± 9.2	33.4 ± 9.1
Median age (IQR) (years)	35.0 (28.0 to 42.0)	31.0 (25.0 to 40.0)	32.0 (26.0 to 40.0)
Age group (years)			
<25	12 (11.4)	56 (21.9)	68 (18.8)
25 to 34	35 (33.4)	101 (39.4)	136 (37.7)
35 to 44	39 (37.1)	63 (24.6)	102 (28.3)
45 to 54	18 (17.1)	34 (13.3)	52 (14.4)
≥55	1 (1.0)	2 (0.8)	3 (0.8)
Country of origin			
Benin	54 (51.4)	125 (48.8)	179 (49.6)
Togo	24 (22.9)	68 (26.6)	92 (25.5)
Nigeria	16 (15.2)	44 (17.2)	60 (16.6)
Ghana	10 (9.5)	14 (5.5)	24 (6.6)
Other	1 (1.0)	5 (1.9)	6 (1.7)
Education^b^			
None	43 (41.4)	75 (29.3)	118 (32.8)
Primary	41 (39.4)	93 (36.3)	134 (37.2)
Secondary (level 1)	13 (12.5)	62 (24.2)	75 (20.8)
Secondary (level 2)	5 (4.8)	19 (7.4)	24 (6.7)
University	2 (1.9)	7 (2.7)	9 (2.5)
Marital status			
Single	29 (27.6)	92 (35.9)	121 (33.5)
Divorced/Separated	59 (56.2)	114 (44.5)	173 (47.9)
Widowed	16 (15.2)	44 (17.2)	60 (16.6)
Married	1 (1.0)	6 (2.3)	7 (1.9)
Gonorrhea^c^			
Positive	6 (5.9)	28 (11.2)	34 (9.7)
Negative	96 (94.1)	222 (88.8)	318 (90.3)
Chlamydia^c^			
Positive	3 (2.9)	14 (5.6)	17 (4.8)
Negative	99 (97.1)	236 (94.4)	335 (95.2)
Active Syphilis^e^			
Negative	105 (100.0)	256 (100.0)	361 (100.0)
Bacterial vaginosis^d^ (Nugent score)			
Normal flora (0 to 3)	11 (10.8)	25 (10.0)	36 (10.2)
Intermediate flora (4 to 6)	29 (28.4)	77 (30.7)	106 (30.0)
Vaginosis (7 to 10)	62 (60.8)	149 (59.4)	211 (59.8)
Candidiasis^d^			
Positive	7 (6.9)	10 (4.0)	17 (4.8)
Negative	95 (93.1)	241 (96.0)	336 (95.2)
*Trichomonas vaginalis* d			
Positive	1 (1.0)	1 (0.4)	2 (0.6)
Negative	101 (99.0)	250 (99.6)	351 (99.4)
Hepatitis B^f^^,^ h			
anti‐HBs positive^h^	55 (49.5)	117 (37.6)	172 (40.8)
anti‐HBc positive[Link]	93 (83.8)	202 (65.0)	295 (69.9)
HBsAg positive	5 (4.5)	14 (4.5)	19 (4.5)
CD4^j^			
˂500 cells/mm^3^	56 (53.3)	–	–
≥500 cells/mm^3^	49 (46.7)	–	–
Viral load^j^			
Undetectable (<40 copies/mL)	3 (2.9)	–	–
Suppressed (<1000 copies/mL)	17 (16.2)	–	–

^a^E‐ART participants were older than Pre‐exposure prophylaxis (PrEP) participants (*p *= 0.005. Student's *t*‐test); ^b^one missing in the E‐ART arm (E‐ART participants were less educated than PrEP participants; *p *= 0.037. chi‐square test with 4 degrees of freedom); ^c^three missing in the E‐ART arm and six missing in the PrEP arm; ^d^three missing in the PrEP arm and five missing in the E‐ART arm; ^e^a case of active syphilis was defined as testing positive for both the SD Bioline rapid test and the RPR test. None of the participants had active syphilis. 4 PrEP participants tested positive on SD Bioline rapid test but their RPR tests were negative. None of the E‐ART participants tested positive on SD Bioline rapid test.; ^f^women positive to anti‐HBs were considered immune to hepatitis B. Women who were anti‐HBc positive and anti‐HBs negative were further tested with the Monolisa‐Biorad HBsAg to detect active hepatitis B infection.; ^g^the denominators used for the E‐ART group was the number of female sex workers (FSWs) with a positive HIV status at screening (N* *= 111) and for the PrEP group all HIV‐negative FSWs at screening (N* *= 311) in order to properly compare HIV‐positive and ‐negative since women with active hepatitis B were excluded from PrEP. ^h^ *p *= 0.028; ^i^ *p *= 0.0002; ^j^CD4 count and viral load evaluated at baseline before initiation of treatment.

### 3.3 Retention

**Figure 2:**
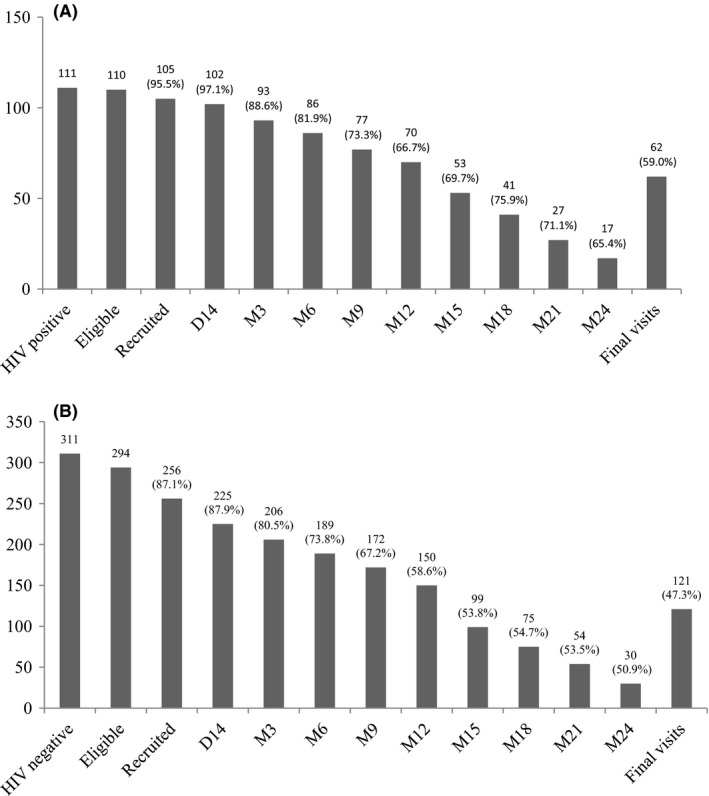
**HIV E‐ART and PrEP cascades among FSWs in the demonstration study in Cotonou, Benin (2014 to 2016)** **(A)** E‐ART cohort **(B)** PrEP cohort. The number of eligible participants represents the number of HIV‐positive for E‐ART (A) and the number of HIV‐negative for PrEP (B). The denominators of proportions through the cascade from day 14 (D14) to month 12 (M12) and final visits (all occurred at M12 or later) were the numbers of participants recruited in the E‐ART and PrEP arms respectively. For visits conducted from D14 to M12, the denominator is the number of participants recruited for E‐ART (n* *= 105) or PrEP (n* *= 256). For visits conducted from M15 to M24, the denominators varied depending on the date of recruitment since recruitment lasted for 12 months and total follow‐up varied from 12 to 24 months. Among E‐ART participants (A), the denominators used from M15 to M24 were 76, 54, 38 and 26 respectively. Among PrEP participants (B), the denominators used from M15 to M24 were 184, 137, 101 and 59 respectively. FSW, female sex workers

E‐ART and PrEP cascades at each follow‐up visit are shown in Figure 2. Among participants recruited early enough to be followed up for two years, retention was not statistically different between the two study groups (65.4% (17/26) vs. 50.9% (30/59) *p *= 0.315). Overall retention at the end of the study was 47.3% (121/256) for PrEP and higher at 59.0% (62/105) for E‐ART (*p *= 0.055). Mean duration (±SD) of follow‐up among all participants was 13.4 ± 7.9 months for E‐ART and 11.8 ± 7.9 months for PrEP. At the end of the study, among FSWs still eligible and living in Cotonou, retention was similar between the two study arms (88.6% (62/70) for E‐ART vs. 78.1% (121/155) for PrEP and *p *= 0.091) (Table S4). None of the participants was completely lost to follow‐up, as those who did not formally withdraw were located by the field workers. The different withdrawal reasons for E‐ART and PrEP participants are detailed in Table S5. Most (61.2% or 109/178) of withdrawals from the study were due to participants moving away from the study site or returning to their country of origin. Withdrawals for such reasons were more frequent among E‐ART than PrEP participants (79.1% (34/43) vs. 55.6% (65/135) *p *= 0.01). Among PrEP participants, eleven pregnancies occurred and two FSWs reported breastfeeding. Those women were terminated from the study since they were no longer eligible (Table S5).

### 3.4 Adherence

**Figure 3:**
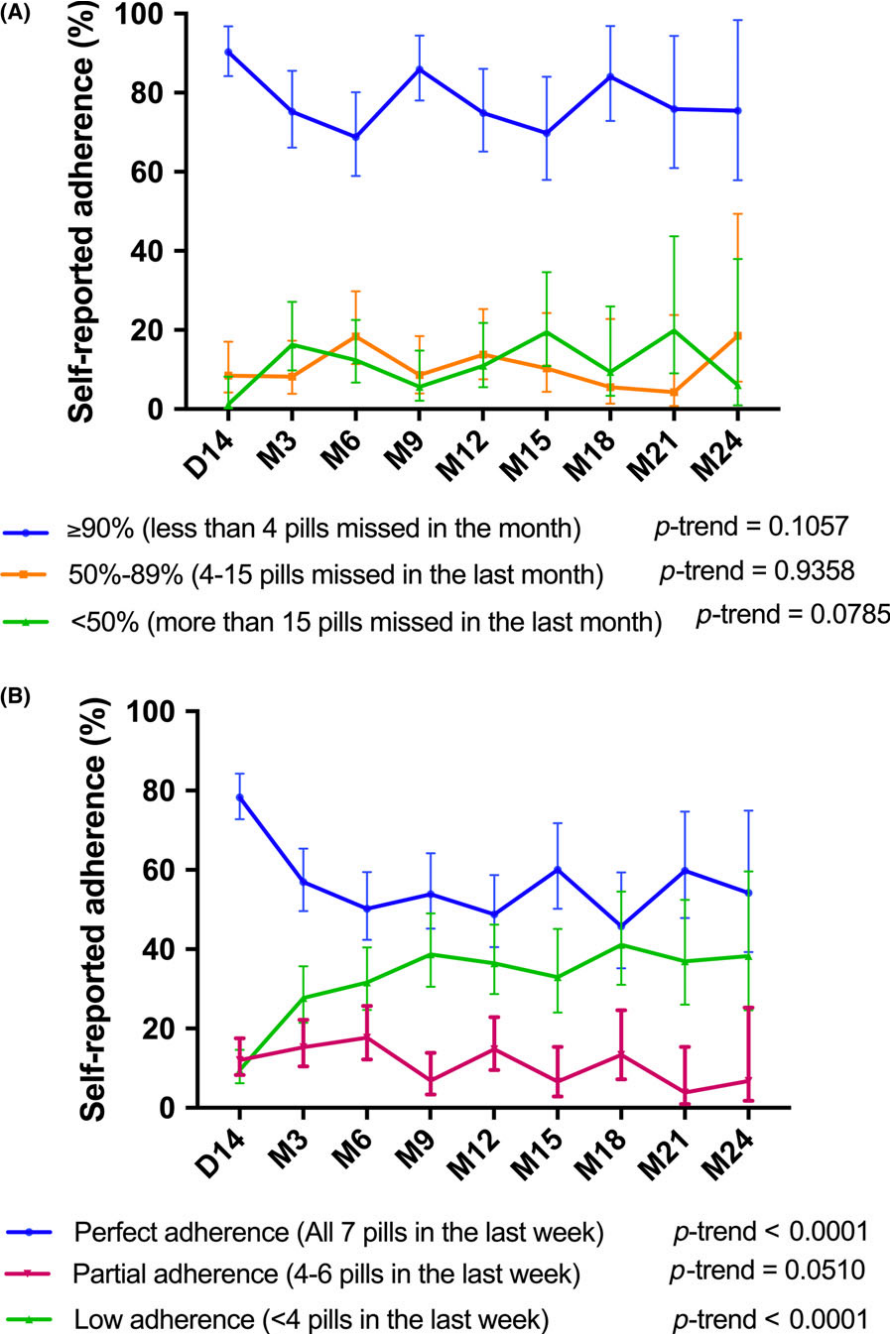
**Self‐reported adherence to E‐ART (A) and PrEP (B) at each scheduled study visit in E‐ART‐PrEP demonstration project Cotonou, Benin (2014 to 2016)** **(A)** E‐ART participants were classified as highly adherent (≥90% or <4 pills missed in the last month), moderately adherent (50% to 89% or 4 to 15 pills missed in the last month), and weakly adherent (<50% or more than 15 pills missed in the last month). Adherence at D14 was estimated by first calculating the number of days between enrolment and the D14 visit, which indicates the number of pills that was supposed to be taken. Second, the number of pills actually taken was divided by the number of days between enrolment and D14. Vertical bars denote 95% confidence intervals (CI). **(B)** PrEP participants reporting taking all pills in the last week were classified as perfectly adherent. If participants declared taking 4 to 6 out of the 7 pills, they were classified as partially adherent. If participants took <4 pills in the week, they were classified as weakly adherent 33. Vertical bars denote 95% CI.

Self‐reported adherence during follow‐up is shown in Figure 3 and Tables S6 to S10. Although no significant trend was observed in highly adherent E‐ART participants during follow‐up (*p *= 0.1057), adherence was significantly lower at their final visit compared to day 14 (75.0% (13/17) vs. 90.5% (76/84), *p *= 0.0205) (Table S7). At the final follow‐up visit, 87.1% (54/62) of the E‐ART participants had a suppressed viral load (<1000 copies/mL), and 67.7% (42/62) had an undetectable viral load (<40 copies/mL) (Table S9). Among PrEP participants (Figure 3B), perfect adherence decreased over time (*p*‐trend: <0.0001). It was 78.4% (156/199) at day 14 and 43.3% (65/150) at the final visit (*p*<0.0001) (Table S12). In addition, FSWs <25 years old were less likely to report perfect adherence compared to those ≥25 years old (Proportion Ratio* *= 0.76, 95% CI: 0.62 to 0.93) (Table S11).

### 3.5 HIV and STI incidence

Among PrEP participants, we observed two HIV seroconversions out of 250.1 person‐years of follow‐up (HIV incidence* *= 0.8/100 person‐years; 95% CI: 0.3 to 1.9/100 person‐years). Both cases were lost to follow‐up for 10 and 11 months respectively and returned to the study with a positive HIV status. They were immediately referred for ART initiation. Gonorrhoea incidence was 12.7/100 (15/117.7 person‐years) and 7.2/100 (18/250.1 person‐years) among E‐ART and PrEP participants (*p *= 0.026) respectively. Chlamydia incidence was 4.2/100 (5/117.7 person‐years) and 4.8/100 (12/250.1 person‐years) among E‐ART and PrEP participants respectively. There were three deaths during the study period (two PrEP and one E‐ART); none of them was related to study participation.

### 3.6 Trends in sexual behaviour

**Figure 4:**
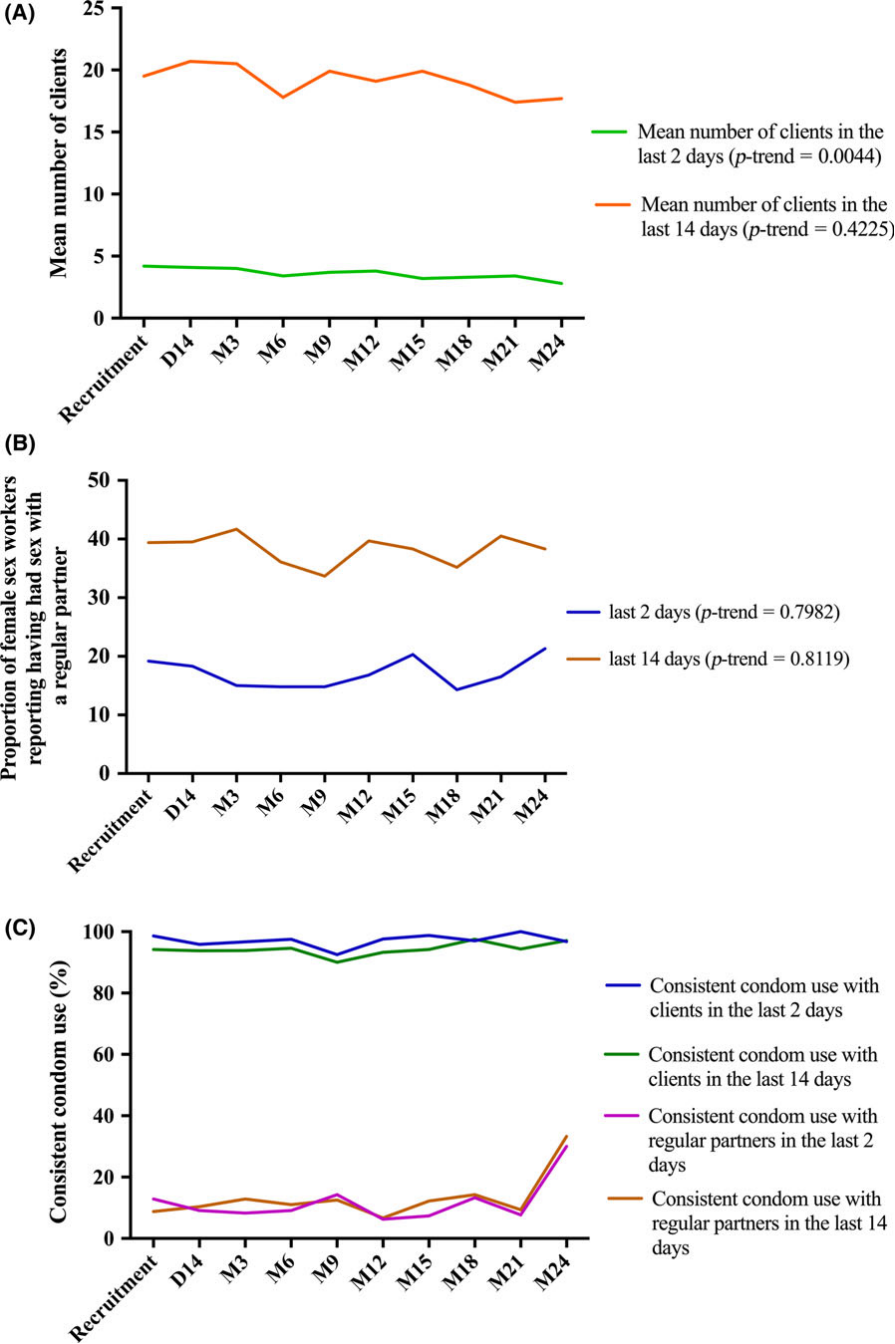
**Trends of sexual behaviour among E‐ART and PrEP participants in the last two or last fourteen days; E‐ART‐PrEP demonstration project, Cotonou, Benin (2014 to 2016)** **(A)** Mean number of clients in the last two or last fourteen days. **(B)** Proportions of female sex workers (FSWs) who reported having had sex with a regular partner in the last two or fourteen days. **(C)** Proportion of FSWs who reported consistent condom use with their sexual partners in the last two or fourteen days. Consistent condom use means having used condoms for all sexual intercourse with the given type of partner over the given time period. No trend in the proportions of self‐reported consistent condom use with clients in the two previous days over the study period (p** *= 0.521). No trend in the proportions of self‐reported consistent condom use with regular partners in the two previous days over the study period (p** *= 0.497). No trend in the proportions of self‐reported consistent condom use with clients in the 14 previous days over the study period (p** *= 0.291). No trend in the proportions of self‐reported consistent condom use with regular partners in the 14 previous days over the study period (p** *= 0.138). *p‐value from generalized estimating equations regression

Figure 4A and Table S14 show that the mean number of clients in the last two days significantly decreased throughout the study for all E‐ART and PrEP participants (*p *= 0.0044) while the mean number of clients in the last fourteen days remained constant (*p *= 0.4225). However, the decrease in the number of clients in the last two days was more pronounced for E‐ART participants (Table S14). The proportion of FSWs reporting having had sex with a regular partner in the last two or fourteen days were low and did not vary during the study (Figure 4B and Table S16). Consistent condom use with clients in the last two and fourteen days was over 90% at all study visits (*p*‐trend* *= 0.521 and *p *= 0.291 respectively) (Figure 4C and Table S17). There was no difference in these trends between E‐ART and PrEP participants (Figure S1 and Tables S17, S18). However, condom use was low (<15% at most visits) with regular sexual partners throughout follow‐up (Figure 4C and Table S18).

## 4 Discussion

In this prospective observational demonstration study, we screened the majority of professional FSWs in the study area for HIV. This is of particular importance as HIV testing coverage is essential to assess E‐ART and PrEP feasibility. The high testing uptake we observed in this target population was expected, since in a study carried out in 2013, 87.1% of FSWs reported having ever been tested for HIV in their lifetime without previous FSW‐specific programmes aiming at increasing testing 19. HIV prevalence, with all the progresses made, was still high in this country characterized by an HIV epidemic largely concentrated among FSWs 20. Uptake of both E‐ART and PrEP was high, but HIV‐positive FSWs were more willing to participate in E‐ART than HIV‐negative FSWs were to participate in PrEP. This situation was predictable given the obvious need for treatment among HIV‐positive women 21. Observed retention rates were arguably low by the end of the study but did not differ between E‐ART and PrEP participants still living in Cotonou. Among PrEP participants, two cases of seroconversions occurred. These two FSWs had been lost to follow‐up for several months and returned to the study with a positive HIV status. Among PrEP participants, adherence was poor and decreased over the course of the study. E‐ART participants reported high and relatively stable adherence throughout the study.

The low HIV incidence found in our study was consistent with results from randomized clinical trials (RCT) and other open‐label studies 13. The fact that seroconversions occurred in FSWs who were off medication for a long period of time clearly emphasize that low PrEP adherence or non‐use in the setting of risk may lead to HIV acquisition 22. In our study, overall adherence to PrEP (47.8% to 78.4%) was low compared to adherence (70% to 95%) found in the demonstration study conducted among FSWs in South Africa (TAPS study) 18, but was consistent with results of RCTs that involved high‐risk women 13, 15, 16. This variability highlights that adherence may be influenced by social and structural factors that should be taken into account in adherence interventions 23. Although PrEP is an effective preventive method among adherent women, it does not need to be lifelong and is only required during the periods of high risk 24, 25. Therefore, adherence should be considered in the context of HIV risk (also known as prevention‐effective adherence) 22, 25.

Prior to the implementation of our demonstration project (2006 to 2014), 48 HIV‐positive FSWs among all FSWs (professional or not) had started E‐ART at the DIST since its availability in the country in 2006 (N. Geraldo, personal communication). As they were well known to the DIST staff members, they were not eligible for the study, which was designed to recruit only treatment‐naive FSWs. This low treatment coverage guided us to recruit all HIV‐positive FSWs regardless of their CD4 count in order to increase treatment coverage and to correspond to the practice that would be put in place for HIV treatment when an E‐ART policy is adopted. There was a good response to treatment among E‐ART participants. Adherence and viral load suppression estimates suggest a relatively good adherence to ART. High adherence to E‐ART in our study was expected given previous results of treatment adherence among FSWs 26 and was consistent with results of the TAPS study 18. The proportion of E‐ART participants with viral suppression at their final visit was 87%. Overall, this could constitutes a good response to treatment although it felt slightly short of the UNAIDS 90% target 27.

As reflected by the E‐ART and PrEP cascades, retention was an issue in both study arms. Lack of retention was mainly due to the high mobility of FSWs. Participants were given the opportunity to refill their medication beyond their monthly supply if they reported that they would be absent for a period of time. For PrEP participants who relocated out of Cotonou, continuous access to the medication was not possible, whereas for E‐ART, availability of medication depended on the HIV treatment policies where the participants relocated. Although retention was low in our study, we found higher rates than the TAPS 18 study where nearly half of the PrEP participants were lost to follow‐up one month after enrolment. Supporting continuous access to PrEP or E‐ART despite the mobility of FSWs is likely to be challenging. Retention issues could be minimized with broad geographical coverage of E‐ART and PrEP programmes in West African countries. Other options include allowing those on stable treatment to collect drugs less frequently or considering medication refill groups to ease drug collection by making such collection possible through peers 23.

We did not observe any evidence of condom migration or risk compensation in either PrEP or E‐ART participants despite concern that the interventions would result in increased sexual risk behaviour. Reported condom use was consistently high with clients but was low with regular partners. Previous findings in this population have shown that 88% of FSWs had used condoms with all clients in the previous week 7, and a relatively low proportion of FSWs had non‐paying partners (28%) 28. This high condom use among FSWs can mostly be attributed to community work on the promotion of the condom and to intervention projects aimed at FSWs and the whole sex work milieu 29. The absence of condom migration was also found in South African FSWs with the TAPS project 18. The HIV incidence we observed was slightly lower but not significantly different from the 1.4/100 person‐years found in a cohort of professional FSWs followed in Benin from 2009 to 2012 30. The STI incidence in that same study 30 was also similar to what we observed in this demonstration project, thus providing additional evidence for the lack of condom migration or risk compensation among FSWs.

Our study has several strengths. We were able to assess testing coverage among FSWs and this is the first step for accessing interventions such as E‐ART and PrEP in a “real‐world” setting. The overall effectiveness of such interventions highly depends on HIV testing uptake 31. Estimating testing coverage as we did is thus an important predictor of E‐ART and PrEP feasibility. We had a more prolonged follow‐up time compared to other demonstration projects 13, 18. Our follow‐up time (12 to 24 months) gave us more opportunity to observe potential seroconversions. We were able to precisely document the reasons why participants were no longer in the study, a clear improvement over loss to follow‐up with undocumented status among study dropouts.

There are a number of caveats to the study. FSWs are highly mobile, and continuous access to the prescribed medications is not guaranteed. In the PrEP arm, pregnant and breastfeeding FSWs were not included due to safety concerns at the time. However, research has shown that there does not appear to be a safety‐related rationale for not prescribing PrEP to pregnant or lactating FSWs who are at continuing risk of HIV infection 32. Given the increase risk of HIV infection in FSWs who become pregnant, FSWs on PrEP who became pregnant could have continued using the medication. We used self‐reported data on adherence, but our results are consistent with findings from RCTs among high risk women 13, and FSWs usually reported when they missed their medications. Sexual behaviours were also self‐reported, but our results were consistent with previous findings in the study population 7, 28.

## 5 Conclusions

This study provides data on key indicators for the integration of E‐ART and PrEP into the HIV prevention combination package already in place for FSWs in Benin. Uptake was good; however, despite intensive efforts to maximize retention and adherence among PrEP users, the suboptimal levels of these key parameters illustrate the difficulties related to the implementation of a PrEP programme among FSWs in Benin. PrEP may be more useful as an intervention at the individual level for adherent FSWs rather than a broader public health intervention. For E‐ART, adherence was not a major issue but retention was suboptimal due to high mobility. E‐ART could be a potential strategy for reducing the HIV burden among FSWs while significantly contributing to HIV prevention at the population level. More research is needed on identifying the FSWs that really need PrEP and determining if intermittent dosing of a PrEP regimen would be more feasible in FSWs in Benin.

## Competing Interests

All authors declare no competing interests.

## Authors’ Contributions

MA, MCB, LB and PV conceived the study. NG, LK, RKK, MB, KD, LG, FC, CL, DA, SD, DMZ, FG, MPG and FAG helped with the study conception. LB, MAG, EMG managed the data and AM, LB, MAG and KG performed data analysis. AM, LB and MA drafted the manuscript. PV, MCB, SD, FAG, KG, MD and MA revised the manuscript for important intellectual content. All authors read and approved the final manuscript.

## Supporting information


**Appendix S1**. Supplementary information _Text, Tables and Figures (DOCX).
**Text S1.** Sample size determination
**Text S2.** Laboratory procedures
**Table S1.** Reasons for reasons for declining E‐ART and PrEP among eligible female sex workers participating in the E‐ART/PrEP demonstration project in Cotonou, Benin
**Table S2.** Comparison of baseline characteristics between female sex workers recruited for E‐ART and FSWs eligible for E‐ART but not recruited in the E‐ART‐PrEP demonstration project in Cotonou, Benin. (2014 to 2016)
**Table S3.** Comparison of baseline characteristics between female sex workers recruited for PrEP and FSWs eligible for PrEP but not recruited in the E‐ART‐PrEP demonstration project in Cotonou, Benin (2014 to 2016)
**Table S4.** Retention among female sex workers recruited in the E‐ART/PrEP demonstration project in Cotonou, Benin
**Table S5.** Reasons for not completing follow‐up till the end of the study among female sex workers participating in the E‐ART/PrEP demonstration project in Cotonou, Benin
**Table S6.** Self‐reported adherence to E‐ART in the last month at each scheduled follow‐up visit in E‐ART‐PrEP demonstration project Cotonou, Benin (2014 to 2016)
**Table S7.** Comparison of adherence (≥90% vs. <90%) to E‐ART at baseline and at final visit in the E‐ART‐PrEP demonstration project Cotonou, Benin (2014 to 2016)
**Table S8.** Comparison of adherence (<50% vs. >50%) to E‐ART at baseline and at final visit in the E‐ART‐PrEP demonstration project Cotonou, Benin (2014 to 2016)
**Table S9.** Viral load by scheduled visit among female sex workers participating in the E‐ART‐PrEP demonstration project in Cotonou, Benin
**Table S10.** Self‐reported adherence to PrEP in the last seven days at each scheduled study visit in E‐ART‐PrEP demonstration project, Cotonou, Benin (2014 to 2016)
**Table S11.** Association between age and perfect adherence (did not miss any pill in the last week) measured by self‐reports among female sex workers participating in the E‐ART‐PrEP demonstration project in Cotonou, Benin
**Table S12.** Comparison of adherence to PrEP in the last seven days at baseline (day 14) and at final visits in the E‐ART‐PrEP demonstration project Cotonou, Benin (2014 to 2016)
**Table S13.** Comparison of adherence to PrEP (≥4 pills vs. all other levels) in the last seven days at baseline (day 14) and at final visits in the E‐ART‐PrEP demonstration project Cotonou, Benin (2014 to 2016)
**Table S14.** Trends in the mean number of clients in the previous two and fourteen days as reported by female sex workers; E‐ART‐PrEP demonstration project, Cotonou, Benin (2014 to 2016)
**Table S15.** Trends in the proportions of female sex workers who reported less than five clients in the last two days and less than 20 clients in the last fourteen days; E‐ART‐PrEP demonstration project, Cotonou, Benin (2014 to 2016)
**Table S16.** Trends in the proportions of female sex workers who reported having had sex with a regular partner in the previous two and fourteen days; E‐ART‐PrEP demonstration project, Cotonou, Benin (2014 to 2016)
**Figure S1.** Trends in sexual behaviours in the last two and fourteen days among female sex workers (FSWs) in Benin demonstration E‐ART‐PrEP project.
**Table S17.** Trends in the proportions of E‐ART and PrEP women who reported consistent condom use (100%) with their clients during the previous two and fourteen days; E‐ART‐PrEP demonstration project Cotonou, Benin (2014 to 2016)
**Table S18.** Trends in the proportions of E‐ART and PrEP women who reported consistent condom use (100%) with their regular sexual partners during the previous two and fourteen days; E‐ART‐PrEP demonstration project, Cotonou, Benin (2014 to 2016)
