# Supplementary material for: Early antiretroviral therapy and daily pre‐exposure prophylaxis for HIV prevention among female sex workers in Cotonou, Benin: a prospective observational demonstration study
Source: J Int AIDS Soc. 2018 Nov 22;21(11):e25208. doi: 10.1002/jia2.25208 (PMC6287093; doi:10.1002/jia2.25208)
Supplement: Supplementary file 1 — Appendix S1. Supplementary information _Text, Tables and Figures (DOCX). Text S1. Sample size determination Text S2. Laboratory procedures Table S1. Reasons for reasons for declining E‐ART and PrEP among eligible female sex workers participating in the E‐ART/PrEP demonstration project in Cotonou, Benin Table S2. Comparison of baseline characteristics between female sex workers recruited for E‐ART and FSWs eligible for E‐ART but not recruited in the E‐ART‐PrEP demonstration project in Cotonou, Benin. (2014 to 2016) Table S3. Comparison of baseline characteristics between female sex workers recruited for PrEP and FSWs eligible for PrEP but not recruited in the E‐ART‐PrEP demonstration project in Cotonou, Benin (2014 to 2016) Table S4. Retention among female sex workers recruited in the E‐ART/PrEP demonstration project in Cotonou, Benin Table S5. Reasons for not completing follow‐up till the end of the study among female sex workers participating in the E‐ART/PrEP demonstration project in Cotonou, Benin Table S6. Self‐reported adherence to E‐ART in the last month at each scheduled follow‐up visit in E‐ART‐PrEP demonstration project Cotonou, Benin (2014 to 2016) Table S7. Comparison of adherence (≥90% vs. <90%) to E‐ART at baseline and at final visit in the E‐ART‐PrEP demonstration project Cotonou, Benin (2014 to 2016) Table S8. Comparison of adherence (<50% vs. >50%) to E‐ART at baseline and at final visit in the E‐ART‐PrEP demonstration project Cotonou, Benin (2014 to 2016) Table S9. Viral load by scheduled visit among female sex workers participating in the E‐ART‐PrEP demonstration project in Cotonou, Benin Table S10. Self‐reported adherence to PrEP in the last seven days at each scheduled study visit in E‐ART‐PrEP demonstration project, Cotonou, Benin (2014 to 2016) Table S11. Association between age and perfect adherence (did not miss any pill in the last week) measured by self‐reports among female sex workers participating in the E‐ART‐PrEP demonstration project i [file JIA2-21-e25208-s001.docx]

**Appendix 1: Supplementary information**

# Early antiretroviral therapy and daily pre-exposure prophylaxis for HIV prevention among female sex workers in Cotonou, Benin: a prospective observational demonstration study

Aminata Mboup, Luc Béhanzin, Fernand A Guédou, Nassirou Geraldo , Ella Goma-Matsétsé , Katia Giguère , Marlène Aza-Gnandji , Léon Kessou , Mamadou Diallo , René K Kêkê , Moussa Bachabi , Kania Dramane , Lily Geidelberg , Fiona Cianci , Christian Lafrance , Dissou Affolabi , Souleymane Diabaté , Marie-Pierre Gagnon, Djimon M Zannou , Flore Gangbo , Marie-Claude Boily , Peter Vickerman, Michel Alary

**Text S1. Sample size determination**

The sample size was defined by using the mapping and enumeration of professional FSWs in the catchment area carried out during “Équité en Santé”, a project conducted from 2012-2016 by the research team of CHU de Québec in Benin. “Équité en santé” created a favorable environment to conduct this E-ART/PrEP study mainly by involving peer educators in recruitment and active community tracking when women were lost to follow-up. The number of professional FSWs in the area of interest was initially estimated at 600. We expected to reach about 500 FSWs and recruit and follow-up at least 90% of the HIV-positive FSWs (n~100) on E-ART and at least 60% of the HIV-negative FSWs (n~250) on PrEP. The study used a field approach that was easily inserted into an ongoing combination prevention intervention, including clinical, behavioural, and structural components, which was implemented in most of the major cities and towns of Benin.

**Text S2. Laboratory procedures**

For HIV and syphilis testing, we used the SD Bioline HIV/syphilis Duo test (Standard Diagnostics Yongin, South Korea). For women who tested positive for HIV, a capillary blood sample collected through finger prick was confirmed with Immunoflow HIV-1/2 test (Core Diagnostics, Birmingham, UK), the standard HIV confirmatory assay used in HIV testing centres in Benin. For women who tested positive for syphilis, venous blood was drawn to identify active syphilis infections using rapid plasma regain (RPR) testing (also provided by Standard Diagnostics). If women tested positive for both the rapid and RPR tests, they were considered as cases of active syphilis and were treated accordingly.

To test for anti-HBc and anti-HBs, we used the Monolisa-Biorad anti-HBc and the Monolisa-Biorad anti-HBs from Biorad (Marnes-la-Coquette, France). Women negative to both tests were considered as susceptible to hepatitis B and were vaccinated with the Euvax vaccine (Sanofi Pasteur, Lyon, France). Women positive to anti-HBs were considered immune to hepatitis B, whereas those who were anti-HBc positive and anti-HBs negative, were further tested with the Monolisa-Biorad HBsAg to detect active hepatitis B infection.

Vaginal swabs were collected for direct microscopy on wet mount for the visualisation of yeasts and *Trichomonas vaginalis*. An additional swab was gram-stained on another slide and examined for abnormal vaginal flora using the Nugent score for the diagnosis of bacterial vaginosis. For *Neisseria gonorrhoeae* and *Chlamydia trachomatis*, cervical swabs were tested using the NG/CT Probetec® assay from Becton Dickenson (Cockeysville, MD, USA). For pregnancy tests we used a simple urine assay from Intec Laboratories (Xiamen, China).

CD4+ T-cell enumeration was performed using the CyFlow Counter (Partec, Germany). Briefly, 50 µl of EDTA-anticoagulated blood were added to 10 µl of monoclonal antibodies. After incubation for 15 minutes, 1 ml of dilution buffer was added and the sample tube attached to the CyFlow Counter for automated counting. Results were expressed using a histogram (CD4+ T-cells/µl). All blood samples for CD4+ T-cell enumeration were processed on the same day the blood was drawn. For viral load testing, nucleic acids isolation was performed using the NucliSens EasyQ equipment. Briefly, amplification and detection were performed by adding a mixture of reagents (primers solution, molecular beacon probes, nucleotides, dithiothreitol, KCl, MgCl_2_) to a purified nucleic acids extract. The mixture was incubated in the NucliSens EasyQ Incubator (bioMerieux, Marcy l’Etoile, France), and the enzyme solution added to each tube. The sealed tubes were then transferred to the NucliSens EasyQ Analyzer (bioMerieux, Marcy l’Etoile, France). Results were expressed as valid (positive or negative) or invalid. When the result was positive, the viral load was calculated (copies/ml). For both tests (CD4+ T-cell counting and HIV viral load), positive and negative quality controls were used.

**Tables and Figures**

**Table S1. Reasons for declining E-ART and PrEP among eligible female sex workers participating in the E-ART/PrEP demonstration project in Cotonou, Benin (2014-2016)**

| **Justification for withdrawal** | **E-ART (N=5)** | **PrEP (N=34)** | **Total (N=39)** |
| --- | --- | --- | --- |
| Does not want to take a pill daily | 0 | 4 | 4 |
| Wants to think more about PrEP | 0 | 4 | 4 |
| Does not perceive self as at risk or see need to take PrEP | 0 | 9 | 9 |
| Afraid of side effects | 0 | 9 | 9 |
| Out of town, province, country* | 5 | 8 | 13 |

*Left Cotonou between the screening and recruitment visits

**Table S2. Comparison of baseline characteristics between female sex workers recruited for E-ART and FSWs eligible for E-ART but not recruited in the E-ART-PrEP demonstration project in Cotonou,Benin (2014-2016)**

| **Characteristics** | **Participants recruited N=105**  **n(%)** | **Participants eligible but not recruited N=5**  **n(%)** | **p-value*** |
| --- | --- | --- | --- |
| Mean age (±SD) (years) | 35.5±8.8 | 27.0 ± 4.2 | 0.034 |
| Median age (IQR) (years) | 35.0 (28.0-42.0) | 30.0 (22.5 – 30.0) |  |
| Age group (years) |  |  | 0.147 |
| < 25 | 12 (11.4) | 2 (40.0) |  |
| 25 - 34 | 35 (33.4) | 3 (60.0) |  |
| 35 -44 | 39 (37.1) | 0 (0.0) |  |
| 45 -54 | 18 (17.1) | 0 (0.0) |  |
| >= 55 | 1 (1.0) | 0 (0.0) |  |
| Country of origin |  |  | 0.394 |
| Benin | 54 (51.4) | 2 (40.0) |  |
| Togo | 24 (22.9) | 3 (60.0) |  |
| Nigeria | 16 (15.2) | 0 (0.0) |  |
| Ghana | 10 (9.5) | 0 (0.0) |  |
| Other | 1 (1.0) | 0 (0.0) |  |
| Education |  |  | 0.493 |
| None | 43 (41.4) | 1 (20.0) |  |
| Primary | 41 (39.4) | 4 (80.0) |  |
| Secondary (level 1) | 13 (12.5) | 0 (0.0) |  |
| Secondary (level 2) | 5 (4.8) | 0 (0.0) |  |
| University | 2 (1.9) | 0 (0.0) |  |
| Marital status |  |  | 0.097 |
| Single | 29 (27.6) | 4 (80.0) |  |
| Divorced/Separated | 59 (56.2) | 1 (20.0) |  |
| Widowed | 16 (15.2) | 0 (0.0) |  |
| Married | 1 (1.0) | 0 (0.0) |  |

*Fisher’s exact and Student’s *t* tests were used to compute the p-values

**Table S3. Comparison of baseline characteristics between female sex workers recruited for PrEP and FSWs eligible for PrEP but not recruited in the E-ART-PrEP demonstration project in Cotonou. Benin (2014-2016)**

| **Caractéristiques** | **Participants recruited N=256 n(%)** | **Participantes eligible and not recruted N=34 n(%)** | **p-value*** |
| --- | --- | --- | --- |
| Mean age (±SD) (years) | 32.5±9.2 | 27.8 ±6.3 | 0.004 |
| Median age (IQR) (years) | 31.0 (25.0-40.0) | 26.0 (23.0 – 32.7) |  |
| Age group (years) |  |  | 0.085 |
| < 25 | 56 (21.9) | 13 (38.2) |  |
| 25 - 34 | 101 (39.4) | 13 (38.2) |  |
| 35 -44 | 63 (24.6) | 8 (23.6) |  |
| 45 -54 | 34 (13.3) | 0 (0) |  |
| >= 55 | 2 (0.8) | 0 (0) |  |
| Country of origin |  |  | 0.311 |
| Benin | 125 (48.8) | 13 (38.3) |  |
| Togo | 68 (26.5) | 13 (38.3) |  |
| Nigériane | 44 (17.2) | 5 (14.7) |  |
| Ghana | 14 (5.5) | 1 (2.9) |  |
| Other | 5 (2.0) | 2 (5.8) |  |
| Education |  |  | 0.276 |
| None | 75 (29.3) | 8 (23.5) |  |
| Primary | 93 (36.3) | 13 (38.2) |  |
| Secondary (level 1) | 62 (24.2) | 7 (20.6) |  |
| Secondary (level 2) | 19 (7.4) | 6 (17.7) |  |
| University | 7 (2.8) | 0 (0.0) |  |
| Marital status |  |  | 0.031 |
| Single | 92 (35.9) | 21 (61.8) |  |
| Divorced/Separated | 114 (44.5) | 8 (23.5) |  |
| Widowed | 44 (17.2) | 4 (11.8) |  |
| Married | 6 (2.4) | 1 (2.9) |  |

*Chi-square and Student’s *t* tests were used to compute the p-values

**Table S4. Retention among female sex workers recruited in the E-ART/PrEP demonstration project in Cotonou, Benin (2014-2016)**

|  | **E-ART** | **PrEP** | **P-value** |
| --- | --- | --- | --- |
| Number of FSWs recruited | 105 | 256 |  |
| Number of FSWs at final visit | 64 | 121 |  |
| Number of seroconversions | - | 2 |  |
| Strict retention rate | 62/105^‡^ (59.0%) | 121/256 (47.3 %) | 0.055 |
| Moved outside Cotonou | 19 | 46 |  |
| Returned to country of origin | 15 | 29 |  |
| No longer eligible in the study | 0 | 22 |  |
| Death | 1 | 2 |  |
| Eligible retention rate* | 62/70 (88.6%) | 121/155 (78.1%) | 0.091 |

^‡^The denominator excludes cases of seroconversion

* The denominator was calculated based on the number of female sex workers still eligible and living in Cotonou at the end of the study.

**Table S5. Reasons for not completing follow-up till the end of the study among female sex workers participating in the E-ART/PrEP demonstration project in Cotonou, Benin (2014-2016)**

| **Justification for withdrawal** | **E-ART (N=43)** | **PrEP (N=135)** | **Total**  **(N=178)** |
| --- | --- | --- | --- |
| Moved out of town, province, country | 19 | 46 | 65 |
| Returned to country of origin | 15 | 29 | 44 |
| Not interested in the study anymore | 7 | 23 | 30* |
| No longer eligible for pregnancy reasons** | - | 11 | 11 |
| Desire to marry | 1 | 6 | 7 |
| Didn't like taking pill every day | - | 4 | 4 |
| Side effects | - | 4 | 4 |
| Not engaged in sex work anymore | - | 3 | 3 |
| Death | 1 | 2 | 3 |
| No longer eligible (breastfeeding) | - | 2 | 2 |
| Seroconversion | - | 2 | 2 |
| Other | - | 2 | 2 |
| Partner’s request | - | 1 | 1 |

* The various reasons listed for those not interested in the study anymore are: size of the pill, preference to use condoms consistently, fear of long-term side effects, did not want to take a pill daily, difficulty of understanding why an HIV negative person should take the same medication as an HIV positive person, fear of discrimination, partners request, long waiting time at the clinic during follow-up visits

**Even though these women were excluded from the PrEP arm, they were told to advise their partners to get tested for HIV. They were also counselled on the importance of consistent condom use with other sexual partners and were regularly followed-up for treatment of any STI.

**Table S6. Self-reported adherence to E-ART in the last month at each scheduled follow-up visit in E-ART-PrEP demonstration project Cotonou, Benin (2014-2016)**

| **Adherence level** | **Visit ranking** | | | | | | | | | **p-trend**^§^ |
| --- | --- | --- | --- | --- | --- | --- | --- | --- | --- | --- |
|  | **D-14***  **N=84**  **n (%)** | **M3**  **N=75**  **n (%)** | **M6**  **N=74**  **n (%)** | **M9**  **N=70**  **n (%)** | **M12**  **N= 65**  **n (%)** | **M15**  **N=47**  **n (%)** | **M18**  **N=34**  **n (%)** | **M21**  **N=25**  **n (%)** | **M24**  **N=17**  **n (%)** |  |
| **≥90% (did not miss more than 3 pills in the last month)** | 76 (90.5) | 57 (76.0) | 51 (68.9) | 60 (85.7) | 49 (75.4) | 33 (70.2) | 29 (85.3) | 19 (76.0) | 13 (76.5) | 0.1057 |
| **50-89% (missed 4-15 pills in the last month)** | 7 (8.3) | 6 (8.0) | 14 (18.9) | 6 (8.6) | 9 (13.9) | 5 (10.6) | 2 (5.9) | 1 (4.0) | 3 (17.6) | 0.9358 |
| **<50% (missed more than 15 pills in the last month)** | 1 (1.2) | 12 (16.0) | 9 (12.2) | 4 (5.7) | 7 (10.7) | 9 (19.2) | 3 (8.8) | 5 (20.0) | 1 (5.9) | 0.0785 |

*Adherence at D-14 was estimated by first calculating the number of days between enrolment and the D-14 visit which indicates the number of pills that is supposed to be taken. Secondly, the number of pills actually taken was divided by the number of days between enrolment and D-14.

^§^GEE regression

**Table S7. Comparison of adherence (≥90% vs <90%) to E-ART at baseline and at final visit in the E-ART-PrEP demonstration project Cotonou, Benin (2014-2016)**

| **E-ART** | **Baseline (D14)**  **% (n/N)** | **Final visits**  **% (n/N)** | **p-value*** |
| --- | --- | --- | --- |
| **Did not miss more than 3 pills in the last month (≥90%)** | 90.5 (76/84) | 75 .0 (48/64) | 0.0205 |
| **< 90% adherence** | 9.5 (8/84) | 25.0 (16/64) |  |

*GEE regression

**Table S8. Comparison of adherence (<50% vs >50%) to E-ART at baseline and at final visit in the E-ART-PrEP demonstration project Cotonou, Benin (2014-2016)**

| **E-ART** | **Baseline (D14)**  **% (n/N)** | **Final visits**  **% (n/N)** | **p-value*** |
| --- | --- | --- | --- |
| **Missed more than 15 pills in the last month (<50%)** | 1.2 (1/84) | 14.1 (9/64) | 0.0118 |
| **> 50% adherence** | 98.8 (83/84) | 85.9 (55/64) |  |

* GEE regression

**Table S9. Viral load by scheduled visit among female sex workers participating in the E-ART-PrEP demonstration project in Cotonou, Benin (2014-2016)**

| **Viral Load** | **Recruitment**  **N=105**  **n(%)** | **M6**  **N=80**  **n(%)** | **M12**  **N=65**  **n (%)** | **M18**  **N=35**  **n(%)** | **M24**  **N=16**  **n (%)** | **Final visits**  **N=62**  **n (%)** |
| --- | --- | --- | --- | --- | --- | --- |
| **Undetectable (<40 copies/mL)** | 3 (2.9) | 52 (65.0) | 46 (70.8) | 19 (54.3) | 14 (87.5) | 42 (67.7) |
| **Suppressed (<1000 copies/mL)** | 17 (16.2) | 59 (73.8) | 55 (84.6) | 27 (77.1) | 14 (87.5) | 54 (87.1) |

**Table S10. Self-reported adherence to PrEP in the last 7 days at each scheduled study visit in E-ART-PrEP demonstration project, Cotonou, Benin (2014-2016)**

|  | **Visit ranking** | | | | | | | | | **p-trend*** |
| --- | --- | --- | --- | --- | --- | --- | --- | --- | --- | --- |
| **Adherence level** | **D-14**  **N=199**  **n (%)** | **M3**  **N=151**  **n (%)** | **M6**  **N=130**  **n (%)** | **M9**  **N=103**  **n (%)** | **M12**  **N=115**  **n (%)** | **M15**  **N=76**  **n (%)** | **M18**  **N=67**  **n (%)** | **M21**  **N=51**  **n (%)** | **M24**  **N=30**  **n (%)** |  |
| **Perfect Adherence (7 pills in the last week)** | 156 (78.4) | 88 (58.3) | 68 (52.3) | 58 (56.3) | 58 (50.4) | 47 (61.8) | 32 (47.8) | 31 (60.8) | 17 (56.7) | <0.0001 |
| **Partial Adherence (4-6 pills in the last week)** | 24 (12.1) | 23 (15.2) | 23 (17.7) | 7 (6.8) | 17 (14.8) | 5 (6.6) | 9 (13.4) | 2 (3.9) | 2 (6.7) | 0.0510 |
| **Low adherence (<4 pills in the last week)** | 19 (9.5) | 40 (26.5) | 39 (30.0) | 38 (36.9) | 40 (34.8) | 24 (31.6) | 26 (38.8) | 18 (35.3) | 11 (36.6) | <0.0001 |

*GEE regression

**Table S11. Association between age and perfect adherence (did not miss any pill in the last week) measured by self-reports among female sex workers participating in the E-ART-PrEP demonstration project in Cotonou, Benin (2014-2016)**

|  | **Visit ranking** | | | | | | | | | **PR***  **(95% CI)** | **p-value*** |
| --- | --- | --- | --- | --- | --- | --- | --- | --- | --- | --- | --- |
|  | **D-14**  **n/N**  **(%)** | **M3**  **n/N**  **(%)** | **M6**  **n/N**  **(%)** | **M9**  **n/N**  **(%)** | **M12**  **n/N**  **(%)** | **M15**  **n/N**  **(%)** | **M18**  **n/N**  **(%)** | **M21**  **n/N**  **(%)** | **M24**  **n/N**  **(%)** |  |  |
| **Perfect adherence among participants <25 years old** | 24/42  (57.1) | 8/22  (36.4) | 7/15  (46.7) | 5/9  (55.6) | 5/11  (45.5) | 5/10 (50.0) | 2/6  (33.3) | 1/6  (16.7) | 1/2  (50.0) | 0.76  (0.62-0.93) | 0.0078 |
| **Perfect adherence among participants ≥25 years old** | 132/157 (84.1) | 80/129  (62.0) | 61/115 (53.0) | 53/94 (56.4) | 53/104  (50.9) | 42/66 (63.6) | 30/61 (49.2) | 30/45 (66.7) | 16/28 (57.1) | 1 |  |

The numbers of participants who have adherence data at day 14 and Months 3, 6, 9, 12,15, 18, 21, 24 are respectively: 199, 151, 130, 103, 115, 76, 67, 51 and 30. The denominators presented in the table are the numbers of participants in the age group.

PR: Proportion ratio. *According to a GEE log-binomial analysis using the observations from all follow-up visits.

**Table S12. Comparison of adherence to PrEP in the last 7 days at baseline (day 14) and at final visits in the E-ART-PrEP demonstration project Cotonou, Benin (2014-2016)**

| **PrEP** | **Baseline (J14)**  **% (n/N)** | **Final visits**  **% (n/N)** | **p-value*** |
| --- | --- | --- | --- |
| **100% (did not miss any pills in the last week)** | 78.4 (156/199) | 43.3 (65/150) | p<0.0001 |
| **< 100% adherence (missed one or more pills in the last week)** | 21.6 (43/199) | 56.7 (85/150) |  |

*GEE regression

**Table S13. Comparison of adherence to PrEP (≥4 pills vs all other levels) in the last 7 days at baseline (day 14) and at final visits in the E-ART-PrEP demonstration project Cotonou, Benin (2014-2016)**

| **PrEP** | **Baseline (D14)**  **% (n/N)** | **Final visits**  **% (n/N)** | **p-value*** |
| --- | --- | --- | --- |
| **4 or more pills taken in the last week** | 90.5 (180/199) | 52.0 (78/150) | p<0.0001 |
| **All other adherence levels** | 9.5 (19/199) | 48.0 (72/150) |  |

* GEE regression

Table S14. Trends in the mean number of clients in the previous 2 and 14 days as reported by female sex workers; E-ART-PrEP demonstration project, Cotonou, Benin (2014-2016)

|  |  | **Recruitment**  **mean (sd)** | **D-14**  **mean (sd)** | **M3**  **mean (sd)** | **M6**  **mean (sd)** | **M9**  **mean (sd)** | **M12**  **mean (sd)** | **M15**  **mean (sd)** | **M18**  **mean (sd)** | **M21**  **mean (sd)** | **M24**  **mean (sd)** |  |
| --- | --- | --- | --- | --- | --- | --- | --- | --- | --- | --- | --- | --- |
|  |  |  |  |  |  |  |  |  |  |  |  | **p-value*** |
| **E-ART** | **Clients in the last 2 days** | 4.3  (4.8) | 4.4  (5.5) | 3.8  (4.6) | 3.2  (4.5) | 3.8  (4.1) | 3.3  (3.9) | 2.2  (3.2) | 2.4  (4.9) | 2.8  (3.7) | 1.9  (2.8) | <0.0001 |
|  | **Clients in the last 14 days** | 19.3  (22.1) | 22.4  (29.8) | 19.4  (24.9) | 18.9  (29.4) | 18.4  (19.9) | 17.6  (20.6) | 15.5  (22.9) | 13.5  (25.3) | 15.9  (24.2) | 15.8  (20.0) | 0.0570 |
| **PrEP** | **Clients in the last 2 days** | 4.1  (4.5) | 4.0  (4.6) | 4.1  (4.3) | 3.5  (3.9) | 3.6  (4.1) | 4.0  (4.9) | 3.7  (5.6) | 3.7  (4.2) | 3.7  (4.9) | 3.2  (3.0) | 0.2893 |
|  | **Clients in the last 14 days** | 19.6  (23.9) | 19.9  (27.8) | 20.9  (21.6) | 17.2  (19.9) | 20.9  (26.8) | 19.9  (22.8) | 22.3  (31.0) | 21.6  (24.0) | 18.1  (22.7) | 18.7  (18.9) | 0.8184 |
| **All** | **Clients in the last 2 days** | 4.2  (4.6) | 4.1  (4.9) | 4.0  (4.4) | 3.4  (4.1) | 3.7  (4.1) | 3.8  (4.6) | 3.2  (4.9) | 3.3  (4.5) | 3.4  (4.6) | 2.8  (2.9) | 0.0044 |
|  | **Clients in the last 14 days** | 19.5  (23.3) | 20.7  (28.5) | 20.5  (22.7) | 17.8  (23.7) | 19.9  (24.4) | 19.1  (22.0) | 19.9  (28.5) | 18.8  (24.6) | 17.4  (23.0) | 17.7  (19.2) | 0.4225 |

*p-value from GEE regression

Table S15. Trends in the proportions of female sex workers who reported less than 5 clients in the last 2 days and less than 20 clients in the last 14 days; E-ART-PrEP demonstration project, Cotonou, Benin (2014-2016)

|  |  | **Recruitment**  **% (n)** | **D-14**  **% (n)** | **M3**  **% (n)** | **M6**  **% (n)** | **M9**  **% (n)** | **M12**  **% (n)** | **M15**  **% (n)** | **M18**  **% (n)** | **M21**  **% (n)** | **M24**  **% (n)** |  |
| --- | --- | --- | --- | --- | --- | --- | --- | --- | --- | --- | --- | --- |
|  |  |  |  |  |  |  |  |  |  |  |  | **p-value*** |
| **E-ART** | **<5 clients in the last 2 days** | 63.8  (67/105) | 67.0 (67/100) | 67.5  (56/83) | 74.4  (61/82) | 59.5  (44/74) | 69.7  (46/66) | 81.2  (39/48) | 86.1  (31/36) | 69.2  (18/26) | 88.2  (15/17) | 0.0024 |
|  | **<20 clients in the last 14 days** | 64.8  (68/105) | 64.9 (63/97) | 71.6  (58/81) | 68.3  (56/82) | 62.2  (46/74) | 66.7  (44/66) | 75.0  (36/48) | 77.8  (28/36) | 73.1  (19/26) | 70.6  (12/17) | 0.2547 |
| **PrEP** | **<5 clients in the last 2 days** | 65.6  (168/256) | 63.7 (135/212) | 59.2 (100/169) | 67.1 (100/149) | 71.3 (87/122) | 68.5 (85/124) | 69.4  (59/85) | 66.7  (46/69) | 73.1  (38/52) | 70.0  (21/30) | 0.2193 |
|  | **<20 clients in the last 14 days** | 69.4  (172/248) | 68.4 (141/206) | 56.8 (96/169) | 64.4 (96/149) | 63.9 (78/122) | 61.5 (75/122) | 67.1  (57/85) | 59.4  (41/69) | 71.2  (37/52) | 66.7  (20/30) | 0.9042 |
| **All** | **<5 clients in the last 2 days** | 65.1  (235/361) | 64.7 (202/312) | 61.9 (156/252) | 69.7 (161/231) | 66.8 (131/196) | 68.9 (131/190) | 73.7 (98/133) | 73.3 (77/105) | 71.8  (56/78) | 76.6  (36/47) | 0.0111 |
|  | **<20 clients in the last 14 days** | 68  (240/353) | 67.3 (204/303) | 61.6 (154/250) | 65.8 (152/231) | 63.3 (124/196) | 63.3 (119/188) | 69.9 (93/133) | 65.7 (69/105) | 71.8  (56/78) | 68.1  (32/47) | 0.4869 |

*p-value from GEE regression

Table S16. Trends in the proportions of female sex workers who reported having had sex with a regular partner in the previous 2 and 14 days; E-ART-PrEP demonstration project, Cotonou, Benin (2014-2016)

|  |  | **Recruitment**  **% (n)** | **D-14**  **% (n)** | **M3**  **% (n)** | **M6**  **% (n)** | **M9**  **% (n)** | **M12**  **% (n)** | **M15**  **% (n)** | **M18**  **% (n)** | **M21**  **% (n)** | **M24**  **% (n)** |  |
| --- | --- | --- | --- | --- | --- | --- | --- | --- | --- | --- | --- | --- |
|  |  |  |  |  |  |  |  |  |  |  |  | **p-value*** |
| **E-ART** | **2 days** | 13.3 (14/105) | 11.0 (11/100) | 15.5  (13/84) | 14.6  (12/82) | 14.9  (11/74) | 18.2  (12/66) | 14.6  (7/48) | 8.3  (3/36) | 11.5  (3/26) | 5.9  (1/17) | 0.2951 |
|  | **14 days** | 28.6 (30/105) | 27 (27/100) | 33.3  (28/84) | 31.7  (26/82) | 32.4  (24/74) | 35.4  (23/65) | 37.5  (18/48) | 27.8  (10/36) | 30.8  (8/26) | 35.3  (6/17) | 0.5872 |
| **PrEP** | **2 days** | 21.6 (55/255) | 21.7 (46/212) | 14.8 (25/169) | 14.9 (22/148) | 14.8 (18/122) | 16.1 (20/124) | 23.5  (20/85) | 17.4  (12/69) | 18.9  (10/53) | 30.0  (9/30) | 0.3470 |
|  | **14 days** | 43.9 (112/255) | 45.5 (96/211) | 45.8 (77/168) | 38.5 (57/148) | 34.4 (42/122) | 41.9 (52/124) | 38.8  (33/85) | 39.1  (27/69) | 45.3  (24/53) | 40.0  (12/30) | 0.6209 |
| **All** | **2 days** | 19.2 (69/360) | 18.3 (57/312) | 15  (38/253) | 14.8 (34/230) | 14.8 (29/196) | 16.8 (32/190) | 20.3 (27/133) | 14.3 (15/105) | 16.5  (13/79) | 21.3  (10/47) | 0.7982 |
|  | **14 days** | 39.4 (142/360) | 39.5 (123/311) | 41.7 (105/252) | 36.1 (83/230) | 33.7 (66/196) | 39.7 (75/189) | 38.3 (51/133) | 35.2 (37/105) | 40.5  (32/79) | 38.3  (18/47) | 0.8119 |

*p-value from GEE regression

**
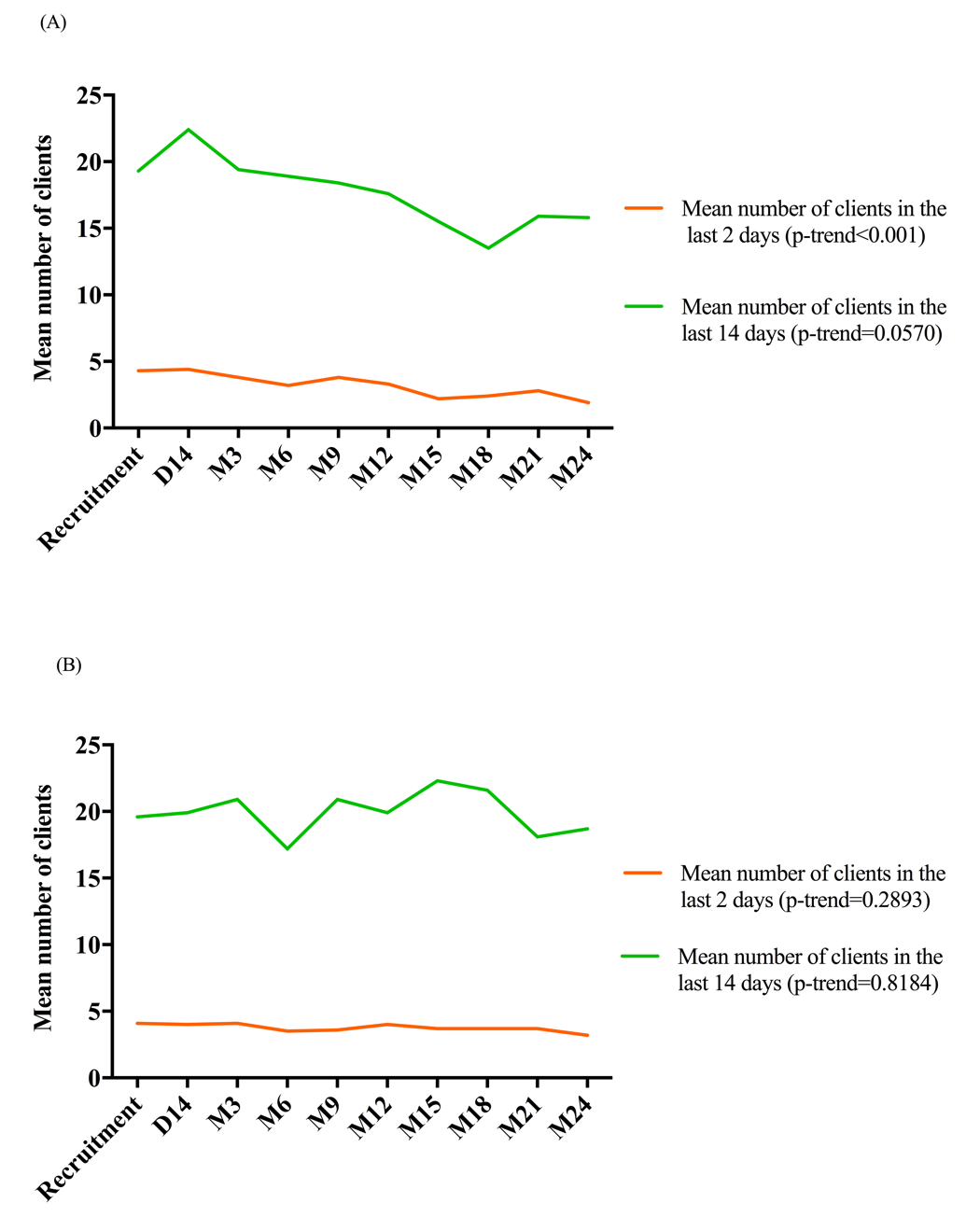
**

**
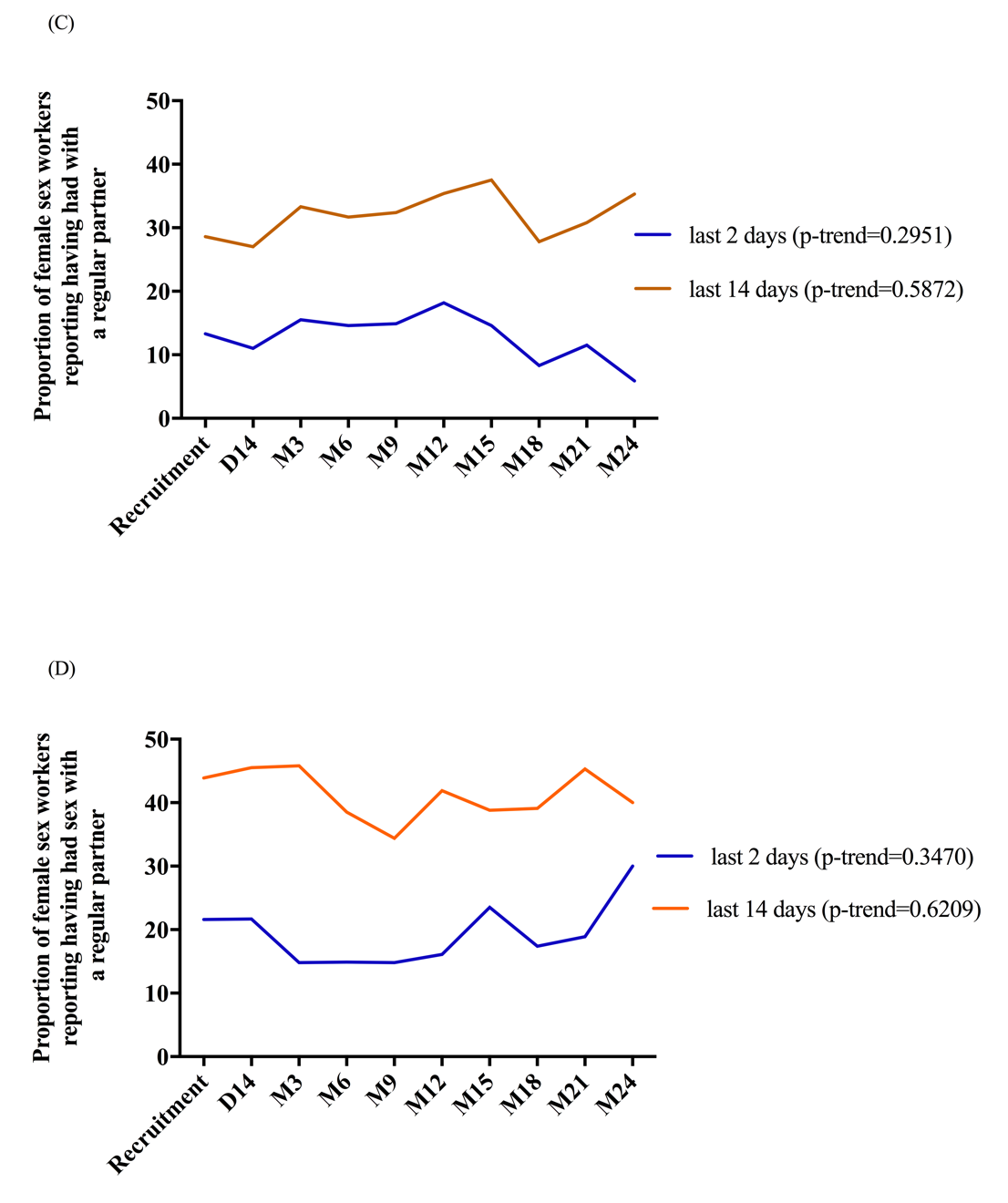
**

**
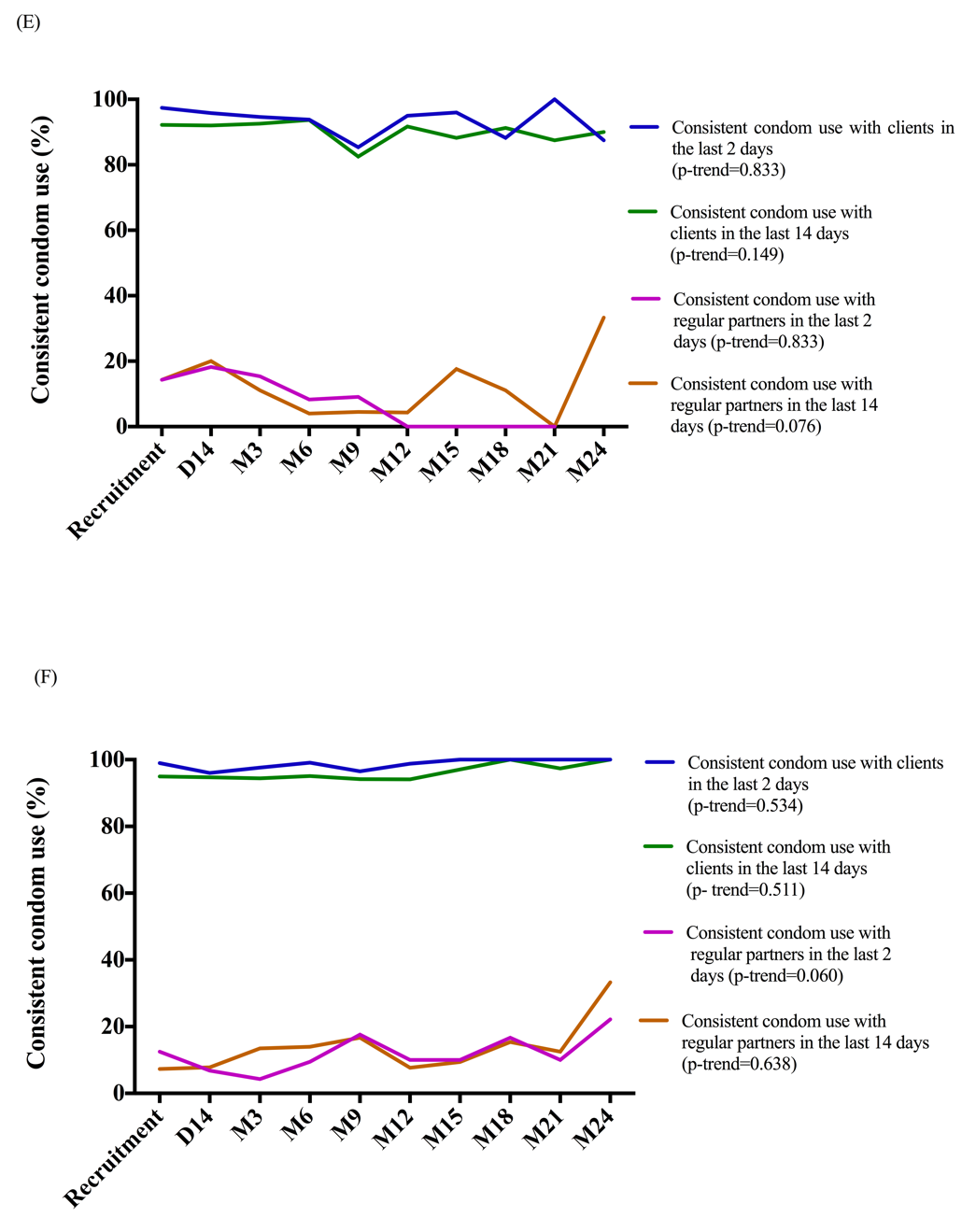
**

**Figure S1. Trends in sexual behaviors in the last 2 and 14 days among female sex workers (FSWs) in Benin demonstration E-ART-PrEP project. (A) Mean number of clients among FSWs under early antiretroviral therapy (E-ART). (B) Mean number of clients among FSWs under HIV pre-exposure prophylaxis (PrEP). (C) Proportion of FSWs under E-ART reporting having had sex with a regular partner. (D) Proportion of FSWs under PrEP reporting having had sex with a regular partner. (E) Self-reported consistent condom use (100%) among FSWs under E-ART. (F) Self-reported consistent condom use (100%) among FSWs under PrEP**

All p-values were computing using GEE regressions

Table S17. Trends in the proportions of E-ART and PrEP women who reported consistent condom use (100%) with their clients during the previous 2 days and 14 days; E-ART-PrEP demonstration project Cotonou, Benin (2014-2016)

|  |  | **Recruitment**  **% (n)** | **D-14**  **% (n)** | **M3**  **% (n)** | **M6**  **% (n)** | **M9**  **% (n)** | **M12**  **% (n)** | **M15**  **% (n)** | **M18**  **% (n)** | **M21**  **% (n)** | **M24**  **% (n)** |  |
| --- | --- | --- | --- | --- | --- | --- | --- | --- | --- | --- | --- | --- |
|  |  |  |  |  |  |  |  |  |  |  |  | **p-value*** |
| **E-ART** | **2 days** | 97.4  (75/77) | 95.8 (68/71) | 94.6  (53/56) | 93.8  (45/48) | 85.4  (41/48) | 95.0  (38/40) | 96.0  (24/25) | 88.2  (15/17) | 100.0 (14/14) | 87.5  (7/8) | 0.534** |
|  | **14 days** | 92.2  (83/90) | 92.0  (81/88) | 92.6  (63/68) | 93.7  (59/63) | 82.5  (47/57) | 91.7  (44/48) | 88.2  (30/34) | 91.3  (21/23) | 87.5  (14/16) | 90.0  (9/10) | 0.630 |
| **PrEP** | **2 days** | 99.0  (198/200) | 96.0  (144/150) | 97.6  (123/126) | 99.1 (109/110) | 96.5  (82/85) | 98.8  (83/84) | 100.0  (57/57) | 100.0  (50/50) | 100.0  (32/32) | 100.0  (23/23) | 0.833*** |
|  | **14 days** | 95.0  (228/240) | 94.7 (177/187) | 94.4 (136/144) | 95.1 (117/123) | 94.2 (97/103) | 94.1 (95/101) | 97.1  (68/70) | 100.0 (56/56) | 97.4  (37/38) | 100.0 (24/24) | 0.060ǂ |
| **All** | **2 days** | 98.6  (273/277) | 95.9 (212/221) | 96.7 (176/182) | 97.5 (154/158) | 92.5 (123/133) | 97.6 (121/124) | 98.8  (81/82) | 97.0  (65/67) | 100.0 (46/46) | 96.8  (30/31) | 0. 521 |
|  | **14 days** | 94.2  (311/330) | 93.8  (258/275) | 93.9  (199/212) | 94.6 (176/186) | 90.0  (144/160) | 93.3 (139/149) | 94.2 (98/104) | 97.5  (77/79) | 94.4  (51/54) | 97.1  (33/34) | 0.291 |

*p-value from GEE regression; **trend from baseline to M18; ***trend from baseline to M12; ǂtrend from baseline to M21

Table S18. Trends in the proportions of E-ART and PrEP women who reported consistent condom use (100%) with their regular sexual partners during the previous 2 and 14 days; E-ART-PrEP demonstration project, Cotonou, Benin (2014-2016)

|  |  | **Recruitment**  **% (n)** | **D-14**  **% (n)** | **M3**  **% (n)** | **M6**  **% (n)** | **M9**  **% (n)** | **M12**  **% (n)** | **M15**  **% (n)** | **M18**  **% (n)** | **M21**  **% (n)** | **M24**  **% (n)** |  |
| --- | --- | --- | --- | --- | --- | --- | --- | --- | --- | --- | --- | --- |
|  |  |  |  |  |  |  |  |  |  |  |  | **p-value*** |
| **E-ART** | **2 days** | 14.3  (2/14) | 18.2  (2/11) | 15.4  (2/13) | 8.3  (1/12) | 9.1  (1/11) | 0.0  (0/12) | 0.0  (0/7) | 0.0  (0/3) | 0.0  (0/3) | 100.0  (1/1) | 0.511** |
|  | **14 days** | 14.3  (4/28) | 20.0  (5/25) | 11.1  (3/27) | 4.0  (1/25) | 4.5  (1/22) | 4.3  (1/23) | 17.6  (3/17) | 11.1  (1/9) | 0.0  (0/8) | 33.3  (2/6) | 0.638ǂ |
| **PrEP** | **2 days** | 12.5  (7/56) | 6.8  (3/44) | 4.3  (1/23) | 9.5  (2/21) | 17.6  (3/17) | 10.0  (2/20) | 10.0  (2/20) | 16.7  (2/12) | 10.0  (1/10) | 22.2  (2/9) | 0.149 |
|  | **14 days** | 7.3  (8/109) | 7.8  (7/90) | 13.5  (10/74) | 14.0  (8/57) | 16.7  (7/42) | 7.7  (4/52) | 9.4  (3/32) | 15.4  (4/26) | 12.5  (3/24) | 33.3  (4/12) | 0.076 |
| **All** | **2 days** | 12.9  (9/70) | 9.1  (5/55) | 8.3  (3/36) | 9.1  (3/33) | 14.3  (4/28) | 6.3  (2/32) | 7.4  (2/27) | 13.3  (2/15) | 7.7  (1/13) | 30.0  (3/10) | 0.497 |
|  | **14 days** | 8.8  (12/137) | 10.4 (12/115) | 12.9 (13/101) | 11.0  (9/82) | 12.5  (8/64) | 6.7  (5/75) | 12.2  (6/49) | 14.3  (5/35) | 9.4  (3/32) | 33.3  (6/18) | 0.138 |

*p-value from GEE regression; **trend from baseline to M9; ǂtrend from baseline to M18
